# Cosmic time calibrator for wireless sensor network

**DOI:** 10.1038/s41598-023-32262-8

**Published:** 2023-04-12

**Authors:** Hiroyuki K. M. Tanaka

**Affiliations:** 1 University of Tokyo, Tokyo, Japan; 2 International Virtual Muography Institute (VMI), Global, Tokyo, Japan

**Keywords:** Aerospace engineering, Civil engineering, Particle physics, Experimental particle physics, Natural hazards, Information theory and computation, Physical oceanography, Seismology

## Abstract

Time synchronization of sensor nodes is critical for optimal operation of wireless sensor networks (WSNs). Since clocks incorporated into each node tend to drift, recurrent corrections are required. Most of these correction schemes involve clients periodically receive RF timing signals from a time server. However, an RF-based scheme is prone to glitches or failure unless operating in a region with almost entirely unobstructed space; hence it only operates well in a limited range of environments. For example, GPS requires open-sky environments. Moreover, the precision of land-based RF schemes is limited to a few micro seconds. In this work, we report on a more versatile and new type of recurrent clock resynchronization scheme called cosmic time calibrator (CTC) and its development and testing. CTC utilizes cosmic-ray muon signals instead of RF signals. Muons are penetrative and continuously precipitating onto the Earth’s surface, and they tend to travel linearly through encountered matter at approximately the speed of light in vacuum. Therefore, muons themselves can periodically transfer the precise timing information from node to node; hence, the performance of the inter-nodal communication device such as Wi-Fi or Bluetooth is minimized/unnecessary for an online/offline WSN analysis. The experimental results have indicated that a resynchronization frequency and precision of 60 Hz and ± 4.3 ns (S.D.) can be achieved. Modelling work of the WSN-based structural health monitoring of aerospace structures has shown that CTC can contribute to the development of new critical and useful applications of WSN in a wider range of environments.

## Introduction

Clock synchronization is a fundamental process required to improve the overall performance of WSNs. Examples of an important developing WSN technology include wearable sensors and the structural health monitoring (SHM) systems, which are designed to monitor small and large structures such as the human body for continuous measurement of human physiological signals^1^, aircraft^2^ and civil structures^3^ (such as bridges, tunnels, buildings, dams, etc.) and has gained considerable attention over the past two decades. For these applications, inter-nodal time synchronization is a prerequisite, but the ideal time synchronization accuracy is varied depending on the target to be monitored: less than 1 s for automation monitoring, less than 1 ms for vibration, less than 1 microsecond for acoustic monitoring, and less than 1 ns for electricity or light propagation^4^. For example, if modal analysis is conducted without knowing local time information, the identified mode shape phase will be inaccurate, possibly falsely indicating structural damage. The maximum node time synchronization error must be below 120 microseconds; otherwise, due to significant modal shape errors, damage detection and localization will become impossible^5^. WSN time synchronization with a precision better than 1 microsecond is required for measuring the time-of-flight of sound^6^, distributing a beamforming array^7^, integrating a time-series of proximity detections into a velocity estimate^8^, etc. In a wired system, nodal synchronization of the data is a trivial problem. However, a wired configuration is not appropriate in many situations (e.g., inside and outside of an airplane cabin).

In the WSN, stable clocks can be incorporated into each sensor node for time synchronization. However, these clocks will have a tendency to drift (during long periods of operation), and this drift is not usually linearly reversible in time. In order to get the maximum benefit from a WSN, resynchronization of these clocks is necessary to ensure that they are accurately aligned on a common time-scale. To achieve this, the resynchronization signals must have specific characteristics to distribute the valid time data from one location to another. Many network synchronization schemes have been proposed over the years, but most of them use the same basic design: clients periodically receive RF resynchronization signals from a time server.

Invariant common time calibration signals guarantee synchronization of the clocks located at sensor nodes. Photons in a vacuum are the most guaranteed invariant entities that can be used for this signal since electromagnetic waves always travel with a consistent speed and direction inside a vacuum. GPS disciplined clocks use RF signals which have almost the same characteristics as photons in a vacuum for common time calibration signals since the majority of the paths traveled by these RF signals are in environments with characteristics close to a vacuum, except for the region near the surface of the Earth (0 km < *H* < 1,000 km). On the other hand, RF signals passing though the atmosphere are affected by many factors such as variations in atmospheric temperature, humidity, electron density, etc. These factors hamper accurate synchronization of time among the sensor nodes. Nevertheless, commercial GPS receivers provide accuracy better than 200 ns relative to UTC^9^. However, GPS requires a clear sky view, which is unavailable in many application areas. In non-open-sky environments (underground, underwater, beneath dense vegetation, and indoors or inside structures such as a cave, a tunnel, an elevator, a bank safe, etc.), GPS signals are unavailable.

When GPS signals are unavailable, NTP (network time protocol)^10^ is used. NTP with Marzullo’s algorithm^11^ can achieve an accuracy of 200 microseconds or better in local area networks under ideal conditions. However, (A) NTP consumes large amounts of network bandwidth, and (B) NTP is not capable of providing the level of precision that a sensor network requires for peak performance^12^. For Berkeley Mote hardware, Hill et al. report 2-microsend precision for receivers within a single broadcast domain^13^. Reference-broadcast synchronization (RBS)^14^ is also a time synchronization technique used with wireless sensor nodes. RBS functions by differentiating the propagation time difference of an electro-magnetic wave for each beacon receiver. It is only sensitive to differences in the time of received messages, and can eliminate NIC (network interface controller) delay. Cena et al.^14^ evaluated RBS in a real system and concluded that it could achieve an accuracy of 3 microseconds. The Precision Time Protocol (PTP) is also available for wireless networks. Yuan et al. reported the accuracy of the PTP-based clock frequency synchronization < 1 microsecond^15^.

Some of the shortcomings of RF signals (for applications such as SHM) can be overcome by using an alternative probe. For measurements in the shallow subterranean and near surface regions of the Earth, cosmic ray muons travel at a speed more invariant than RF signals in the altitude range (−1 km (underground or underwater) < *H* < 10 km); muons are the most numerous particles in this region, and they travel in the air and water faster than light. Thus, in this region, cosmic ray muons can produce more invariant common time calibration signals than RF signals. Furthermore, due to the penetrative nature of cosmic-ray muons, the muon signals are available in environments which are inaccessible to RF signals or which restrict the transmission of RF signals. Tanaka^16^ proposed Cosmic Time Synchronizer (CTS) to synchronize the local clocks located within the shower disks of extended air showers (EAS). This technique is useful when the size of the area to be synchronized is an order of ~ km^2^. However, due to the uncertainties in the total track lengths of the shower particles, the synchronization accuracy was limited to 100 ns.

The cosmic time calibration (CTC) method proposed in this work realizes accurate resynchronization of local clocks by offering a theoretical accuracy of sub-nanosecond in principle although the applicable spatial range (up to tens of meters) is smaller than CTS. While CTS utilizes the distant simultaneity of arrival timings of air shower particles, CTC utilizes the muon's time of flight. Moreover, muons themselves distribute time to each node, therefore it is not necessary to include standard communication devices between sensor nodes for time synchronization. Since CTC uses a natural and abundant particle probe, the cosmic ray muon, as a signal capable of great temporal accuracy even after passing through a layer of steel, CTC can realize resynchronization of sensor nodes located inside environments where optimal functioning of other devices or concepts fail (e.g., time synchronization between inside a metal made cabin and a metal made airplane wing). For the purpose of online (real-time) monitoring, the CTC sensors need to share the inter-nodal timing data in a real-time manner via a certain inter-nodal communication device (Wi-Fi, blue tooth, ZigBee, etc.), but since this device is not used for time synchronization, it doesn't have to be fast. For the purpose of the offline analysis, any inter-nodal communication device can be removed; hence it is anticipated that CTC has a wide range of applications to WSNs. The concept and principle of CTC will be described in the following sections, and the CTC results, based on the test experiments, and its application to SHM of an aircraft will be reported as an example.

## Results

### Cosmic synchronization of local time

**Figure 1:**
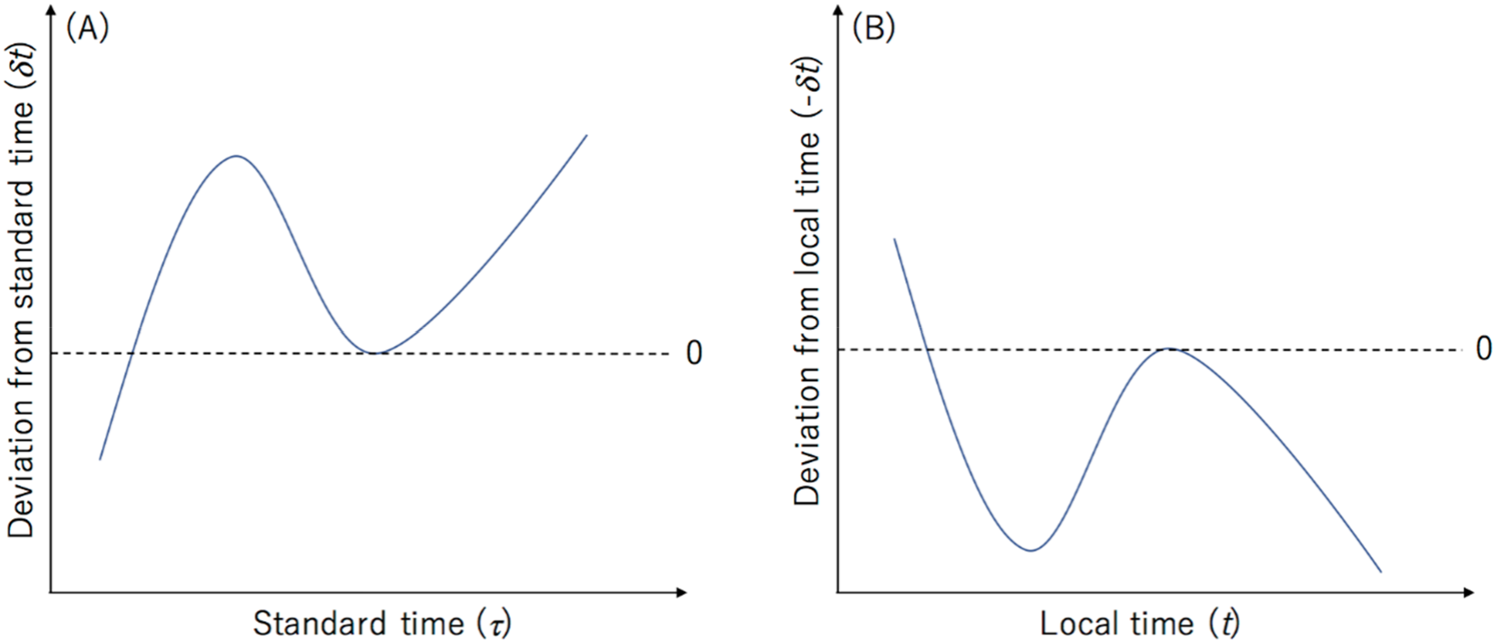
Relationship between local time (*t*) and standard time ($\tau$). While *t* can be timestamped by the standard clock (**A**), $\tau$ can be timestamped by the local clock (**B**). The dashed lines indicate $\delta$ *t* = 0.

Conducting a synchronization of local time (*t*) requires *t* to be resolved with reference to standard time ($\tau$). The value of *t* generally deviates from $\tau$, and this deviation ($\delta$ *t*) can be presented on a 2-dimensional plane as a function of $\tau$ (Fig. 1A). Inversely, $\tau$ can be plotted as a function of *t* (Fig. 1B).

CTC uses cosmic ray muons as calibration signals to correct *n* local clocks associated with *n* sensors which are components of a WSN. One of these *n* local clocks is defined as the standard clock (labeled as Clock 0) and its successive *n* − 1 clocks that are sequentially named here as Clock *i* (*i* = 1, 2,… *n* − 1). The main components for CTC are the *n* non-directionally-sensitive particle detectors (Detector 0 and Detector *i*) which are associated with Clock 0 and Clock *i*. The hardware configuration of CTC will be described in the following section. The times measured by Clock 0 and Clock *n* are respectively labeled with $\tau$ and *t*.

The mass of a muon is 105 MeV at rest, and at sea level, cosmic ray muons are almost all relativistic (typically 3 GeV)^17^ and this characteristic is crucial to the success of the CTC’s synchronization of local clocks. Most cosmic ray muons have a velocity close to the speed of light and thus a $\gamma$ is much larger than 1. Cosmic ray muons are generated near the tropopause as a result of interactions between primary cosmic rays and atmospheric nuclei; however, due to this relativistic nature, the muons' decay lengths ($\lambda$) are extended such that:

1 $$\lambda = \gamma c\tau_{{{\text{DECAY}}}} ,$$

where $\tau$_DECAY_ = 2.2 $\upmu$s is the muon's decay constant, and $\gamma$ is the Lorentz factor. Consequently, they can travel more than 15 km before decaying. The cosmic-ray muon's heavy mass and their relativistic nature enable us to use muons for many purposes including: positioning^18^, navigation^19^ and timing^16^ (PNT) signals and also as radiographic probes to visualize volcanoes^20^, past earthquakes^21^, cultural heritage^22^, tsunami^23^, and tropic cyclones^24^.

The muon's velocity is almost constant since it has a very small dependence on $\gamma$ (for $\gamma$ >  > 1) such that:

2 $$v = \, (c^{{2}} - c^{{2}} \gamma^{{ - {2}}} )^{{{1}/{2}}} .$$

Therefore, it is reasonable for us to approximate the time (*T*_*i*_) required for cosmic-ray muons to travel a distance between Detector 0 and Detector *i* (*D*_*i*_) to be:

3 $$T_{i} = D_{i} c^{{ - {1}}} ,$$

as long as they are relativistic ($\gamma$ >  > 1). Consequently, if a cosmic-ray muon passes through Detector 0 and Detector *i*, the time measured by Clock *i* (*t*_*i*_) is:

4 $$\begin{aligned} t_{i} (\tau ) & = \tau + \delta t(\delta ) + T_{i} \\ & = \tau + D_{i} c^{{ - {1}}} , \\ \end{aligned}$$

$\delta$ *t*($\tau$) comes from the relative drift between Clock 0 and Clock *i*. In Eq. (4), the only unknown parameter is $\delta$ *t*($\tau$). The muon coincidence frequency (${f}_{\upmu }$), and ${f}_{\upmu }$ at the detector is expressed as:

5 $$f_{{\upmu }} = {\Phi }_{i} S_{i} ,$$

where *S*_*i*_ is the effective areas of Detector *i*, and $\Phi$_*i*_ is the muon flux integrated over the solid angular region ($\Omega$_*i*_) formed by Detector 0 and Detector *i* and the energy range between the cutoff energy (*E*_c_) and infinity:

6 $${\Phi }_{i} = \mathop \smallint \limits_{{\phi_{0} }}^{{\phi_{0} + {\Delta }\phi }} \mathop \smallint \limits_{{\theta_{0} }}^{{\theta_{0} + {\Delta }\theta }} \mathop \smallint \limits_{{E_{c} }}^{\infty } I\left( {E,\theta , \phi } \right)dEd\theta d\phi ,$$

In Eq. (6), *I* (*E,* $$\theta$$, $$\phi$$) is the zenith-angular dependent muon intensity^17^. The angular region defined between $$\phi$$_0_ and $$\phi$$_0_+$$\Delta$$$$\phi$$, and $$\theta$$_0_ and $$\theta$$_0_+$$\Delta \theta$$ are respectively small zenith and azimuth angles formed by Detector 0 and Detector *i*. Detector *i* as well as Clock *i* can be concealed inside a thick layer of material, and the value of *E*_c_ depends only on the thickness (*d* gcm^−2^) of this concealing material since the muon range is almost unaffected by the elemental component of materials.

However, in practice, $\tau_{\upmu }$ and $t_{{\upmu i}}$ must be carefully scrutinized since it is possible to generate “false timestamps” which do not represent a successful detection of resynchronization signals and may adversely affect the accuracy of each correction. When a coincident event is observed between Detector 0 and Detector* i*, timestamps $\tau_{\upmu }$ $t_{{\upmu i}}$ are respectively issued for Clock 0 and Clock *i.* This timestamp issuing frequency depends on $f_{\upmu }$ . The probability of generating “false timestamps” will now be described. The single muon count rate is defined as the count rate of the open-sky muons with one detector. This rate is an order of 10^2^ m^2^s^−1^ which is generally much larger than the coincidence rate ($f_{\upmu }$)^17^. Considering this large gap between $f_{\upmu }$ and the single muon count rate, the accidental coincidence rate (*f*_ACCIDENT_) has to be evaluated. In proportion to the coincidence time window (*T*_W_), *f*_ACCIDENT_ increases such that:

7 $$f_{{{\text{ACCIDENT}}}} = \, 2\Phi^{2} S_{0} S_{i} T_{w} ,$$

where $\Phi$ is defined as the single muon count rate per unit area which is ~ 10^2^ m^−2^ s^−1^. On the other hand, |$\delta$ *t*($\tau$)| gradually develops as time goes by. In other words, the clock drifts. Consequently, *t*_*i*_($\tau$) could be much larger than $\tau$ + $\delta$ *t*($\tau$), i.e., eventually, [$\tau$ + $\delta$ *t*($\tau$)]-*t*_*i*_($\tau$) >  > *T*_w_. Therefore, if *T*_W_ is too short, coincident events may not be defined between $\tau_{\upmu }$ and $t_{{{\mu i}}}$. To achieve the optimal value of *T*_W_, we need to adapt the set to be:

8 $$\delta t(\tau ) \, < < T_{{\text{W}}} < < { 1}/{2}f_{{{\text{ACCIDENT}}}} ({\Phi }^{2} S_{0} S_{i} )^{ - 1} .$$

The resynchronization error rate is defined by* f*_ACCIDENT_ $f_{\upmu }$^−1^.

### Cosmic resynchronization device

**Figure 2:**
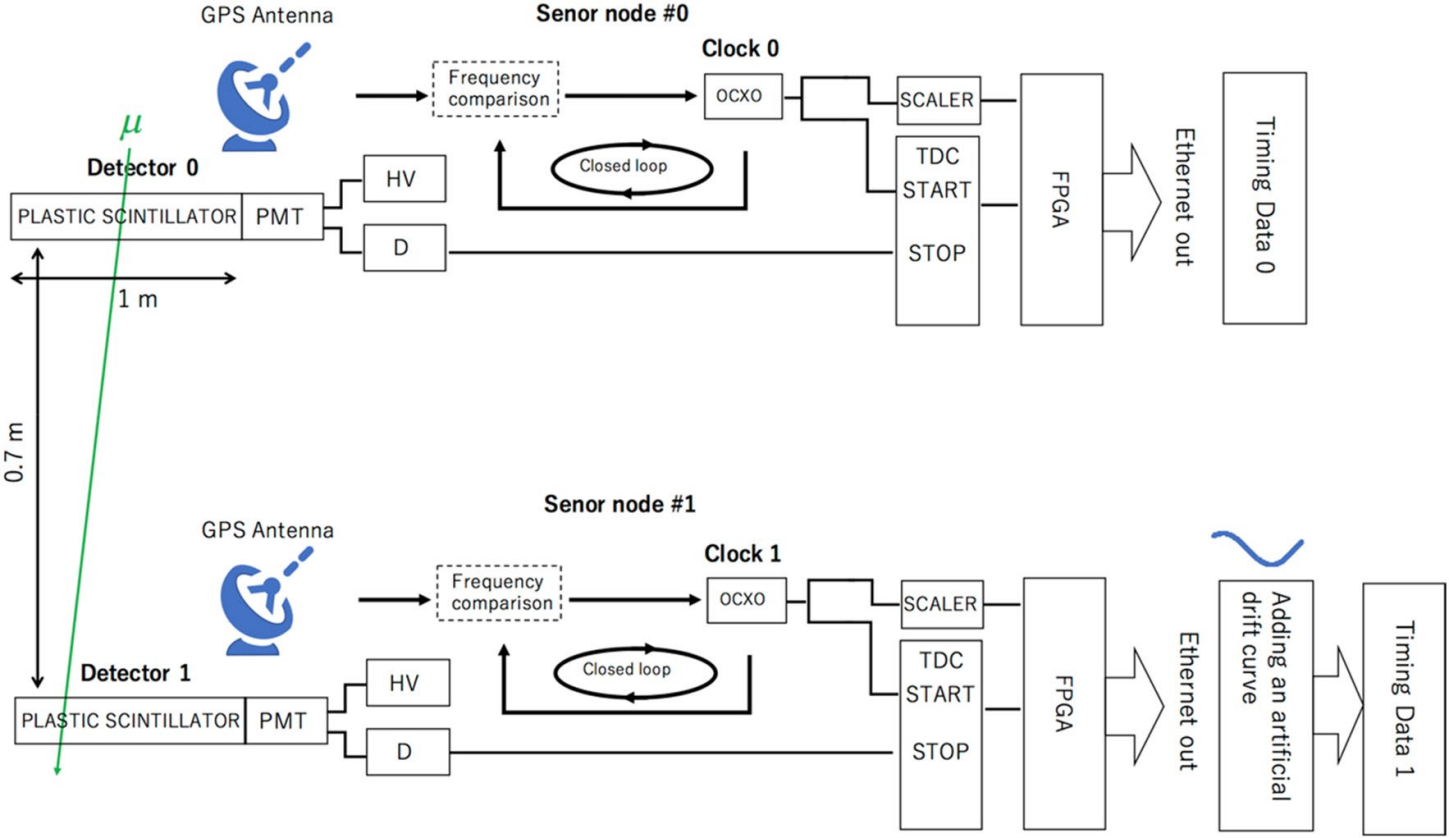
Block diagram of the CTC setup. The CTC Module 0 (top) and the CTC Module 1 (bottom) were setup with a vertical interval of 0.7 m. An artificial drift curve was added to the Clock 1 output. Timing Data 0 and Timing Data 1 were compared to check if this drift curve can be accurately reproduced.

In this section, the configuration of the experimental setup is described. This setup is not compatible with a WSN application, and was considered only to demonstrate the working principle of the CTC method. The design of a compact setup suitable for WSN applications is beyond the scope of the present paper. The current CTC demonstration was conducted with 2 CTC modules (CTC Module 0 and CTC Module 1) associated with the sensor node #0 and the sensor node #1. Each CTC module consists of Detector 0, Detector 1, Clock 0, Clock 1, and the associated electronics. In order to acquire higher statistics for demonstration, each detector utilized in the current work consists of 1 × 1 m^2^ square-shaped plastic scintillators. Detector 0 is vertically displaced from Detector 1 with a vertical interval of *D*_1_ = 70 cm. Photomultiplier tubes (PMT) (Hamamatsu R7724) are connected to each corner of each detector via an acrylic light guide unit. Each detector is equipped with electronics that consist of high voltage supplies (HV), discriminators, scaler electronics and a time to digital converter (TDC) with a time resolution of 27 ps. The absolute time interval (*T*) between the moment when the *n*th signal is outputted from Detector 0 and the moment when the (*n* + 1)th signal is outputted from Detector 0 is measured with a GPS disciplined oscillator (GPS-DO) (Trimble Thunderbolt GM200) and TDC in the following way. A scaler electronics unit counts the number of 10 MHz signals outputted from GPS-DO. Also, these 10 MHz signals, which are outputted from GPS-DO, are divided and fed into a TDC as a start signal. A signal outputted from Detector 0 is fed into a TDC as a stop signal to measure $$\Delta$$$$\tau$$. Detector 1 is equipped with the same electronics. The time offset $$\delta$$ *t*($$\tau$$) between Clock 0 and Clock 1 was reset at the beginning of the measurements by feeding simultaneous signals into the TDC 0 and TDC 1, and the time sequence of $$\tau_{\upmu }$$ and $$t_{{\upmu i}}$$ are recorded at each detector. *T*_w_, was changed within the range between 100 ns and 1 microsecond to compare and identify the time ($$\tau$$ and *t*_1_) when a cosmic-ray muon that has passed through Detector 0 also passes through Detector 1. With the current CTC system, Clock 1 is also coordinated by GPS in order to evaluate its precision. The block diagram of the current setup is shown in Fig. 2. This GPS time data (Clock 1 data) was treated as the standard time ($$\tau$$) to compare with the following drift effect (*f*($$\tau$$)) that was artificially added to $$\tau$$ in the post-processing process such that:

9 $$\delta t(\tau ) = C[{\text{sin}}(\tau /A) + {\text{cos}}(\tau /B)] - D,$$

to validate and confirm the resynchronization results, where the parameters *A*, *B*, *C*, and *D* are respectively fixed to be 5 × 10^14^, 7 × 10^13^, 1 × 10^6^, and 1,366,546, and $\tau$ is in units of ps.

In this work, oven-controlled crystal oscillators (OCXOs) were used for these local clocks. For the purpose of demonstration of this system, a 1-cm thick steel plate was inserted between Detector 0 and Detector 1 to simulate a CTC configuration inside a metal box. The only calibration signals used in this demonstration were cosmic ray muons. There was no inter-nodal communication between Detector 0 and Detector 1 during the measurements. After the measurements, the data recorded at Detector 0 and Detector 1 were retrieved and the history of Clock 1 was retrodicted.

### Resynchronization procedure

In this section, the resynchronization procedure is explained. In reality, since muons can hit any location within the detector area, the muon's travel distance between Detector 0 and Detector *i* will tend to vary as a function of time. Furthermore, travel distance of the photons in the scintillator ($\lambda$_*i*_) will also tend to vary as a function of time since the distance between the PMT and the muon's hitting point within the scintillator varies for each event. Therefore, in reality, Eq. (4) is modified to:

10 $$t_{i} (\tau ) = \tau + \delta t(\tau ) + D_{i} (\tau )c^{{ - {1}}} + \lambda_{i} (\tau )c_{\nu }^{{ - {1}}} ,$$

where $c_{\nu }$ is the speed of light in the material with a refractive index of $\nu$. The maximum time and the minimum values of [*D*_*i*_($\tau$)*c*^−1^ + $\lambda$_*i*_($\tau$)$c_{\nu }$^−1^] are respectively:

11-1 $$[D_{i} (\tau )c^{{ - {1}}} + \lambda_{i} (\tau )c_{\nu }^{{ - {1}}} ]_{{{\text{MAX}}}} = (D_{i}^{{2}} + {2}^{\prime } W_{i}^{{2}} )^{{{1}/{2}}} c^{{ - {1}}} + {1}.{4}W_{i} c_{\nu }^{{ - {1}}}$$

11-2 $$[D_{i} (\tau )c^{ - 1} + \lambda_{i} (\tau )c_{\nu }^{ - 1} ]_{{{\text{MIN}}}} = D_{i} c^{ - 1}$$

where $c_{\nu }$ = *c*/1.49 for a plastic scintillator. Here, temporal fluctuations in conversion times from scintillation photons to TDC time spectrum are small (~ 1 ns) and were neglected in the current discussion. In the current experimental configuration, the values for [*D*_*i*_($\tau$)*c*^−1^ + $\lambda$_*i*_($\tau$)$c_{\nu }$^−1^]_MAX_ and [*D*_*i*_($\tau$)*c*^−1^ + $\lambda$_*i*_($\tau$)$c_{\nu }$^−1^]_MIN_ are respectively ~ 12.2 ns and ~ 2.3 ns.

**Figure 3:**
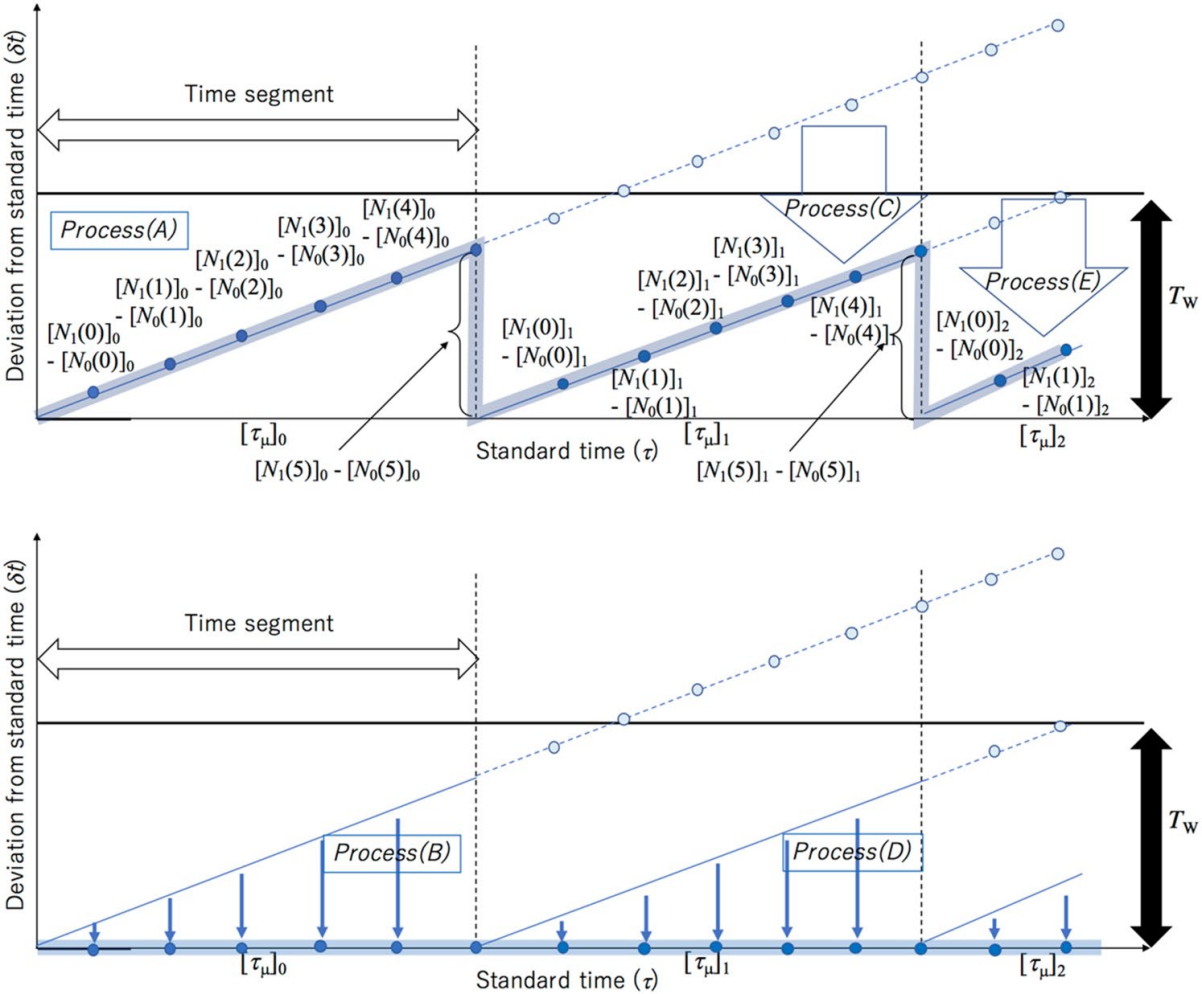
Procedure of the resynchronization process. Blue circles indicate the discrete local timelines that are represented by the clock pulse counts. Filled and blank blue circles respectively indicate the discrete local timelines before and after calibration. Bold blue lines in the bottom panel indicate the recalibrated timeline. Processes (**A**)–(**E**) are indicated in the top panel and the bottom panel, respectively. In this specific case, *i* = 1 and *n*_0_ = 5.The notations of the symbols in this figure are described in the main text.

**Figure 4:**
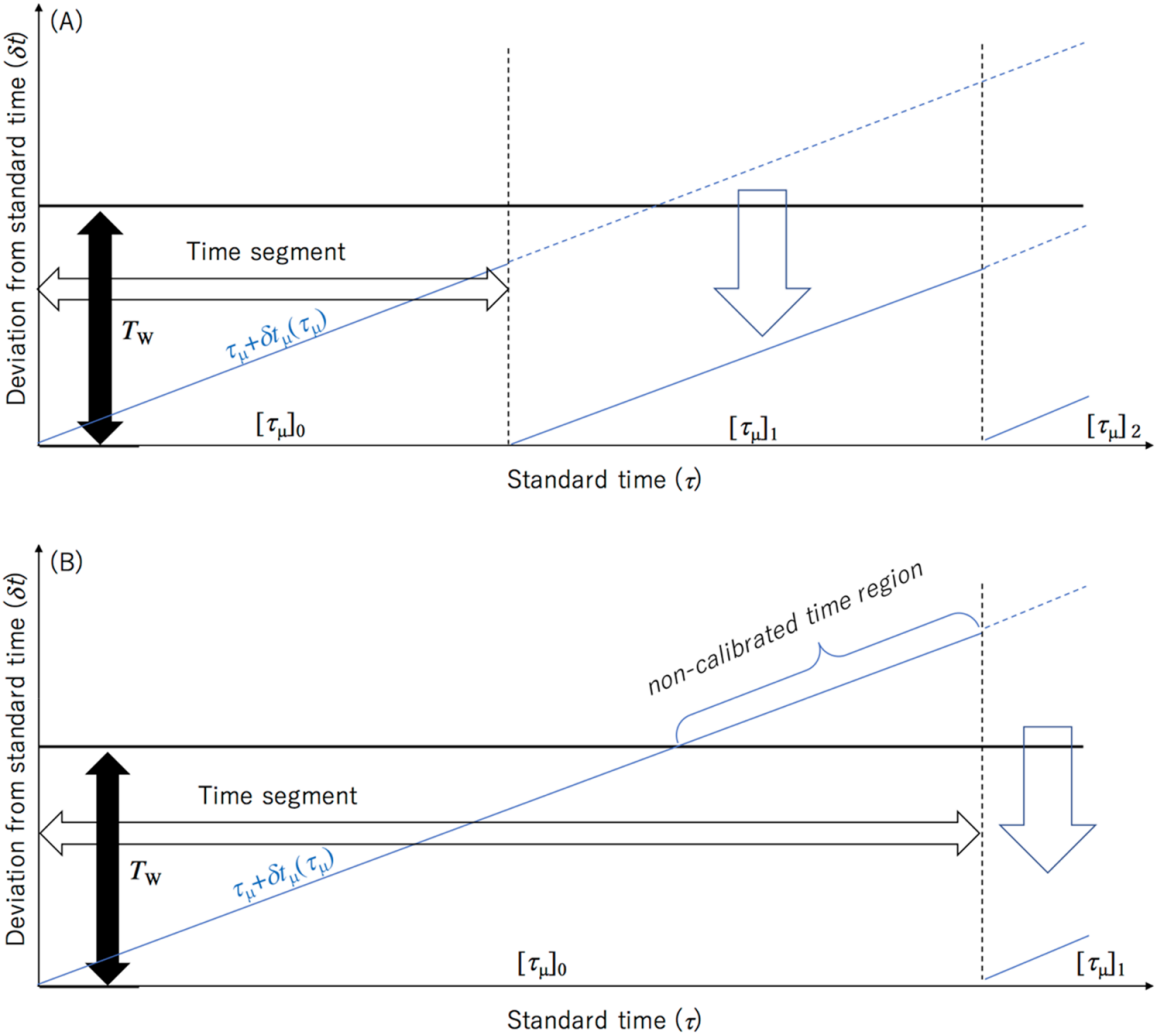
Principle of the resynchronization process. Blue lines indicate the local timelines of the client sensors. Solid and dashed blue lines respectively indicate the timelines before and after calibrations. Black and white box arrows respectively indicate the coincidence time window and the time segment. The notations of the symbols in this figure are described in the main text. The calibration processes are indicated by blue box arrows. If the time segment is sufficiently short, the entire local timelines can be calibrated (**A**). However, if the time segment is longer and if the local timelines exceed the coincidence time window (*T*_W_), part of the local timelines cannot be calibrated as indicated by "non-calibrated time region" in (**B**).

The following procedure describes the resynchronization process. This resynchronization process is also summarized in Fig. 3. A sequence of the timestamps is divided into many short time segments so that the local clock's drift ($\delta$ *t* ($\tau$)) does not exceed *T*_W_ (Fig. 4A,B) so that this resynchronization process can treat only coincidence events.

(A) A sequence of the timestamps, $\tau_{\upmu }$ and $t_{{\upmu i}}$ is divided into many time segments so that $\delta$ *t* ($\tau$) is sufficiently shorter than *T*_W_ within the length of the time segment: [$\tau_{\upmu }$]_*k*=0,1,2,…_ and [$t_{{\upmu i}}$]_*k*=0,1,2,…_, where *k* is the segment identification (SID) number of these segments. Since [$\tau_{\upmu }$]_*k*_ and [$t_{{\upmu i}}$]_*k*_ are respectively expressed as the number of the clock pulse counts: [*N*_0_(*n*)]_*k*_ and [*N*_*i*_(*n*)]_*k*_, [*N*_0_(*n*)]_*k*_ and [*N*_*i*_(*n*)]_*k*_ are compared within the coincidence time window, *T*_W_, to find coincidence events. *n* is the coincidence identification (CID) number of the coincidence events. The length of these time segments must be sufficiently shorter than the timescale which ends when $\delta$ *t*($\tau$) exceeds *T*_W_.

(B) The difference in the pulse counts between Clock 0 and Clock *i* {[*N*_*i*_(*n*)]_0_ − [*N*_0_(*n*)]_0_} is subtracted from all elements of the numerical sequence within the first time-segment of Detector *i*. After this process, the following numerical sequence is generated in the first time-segment:

12 $$\begin{gathered} \left\{ {0, \, \left[ {N_{i} \left( 0 \right)} \right]_{0} - \, \left\{ {\left[ {N_{i} \left( 0 \right)} \right]_{0} - \, \left[ {N_{0} \left( 0 \right)} \right]_{0} } \right\}, \, \left[ {N_{i} \left( {1} \right)} \right]_{0} - \, \left\{ {\left[ {N_{i} \left( {1} \right)} \right]_{0} - \, \left[ {N_{0} \left( {1} \right)} \right]_{0} } \right\}, \ldots } \right\} \hfill \\ = \left\{ {0, \, \left[ {N_{0} \left( 0 \right)} \right]_{0} , \, \left[ {N_{0} \left( {1} \right)} \right]_{0} , \ldots } \right\} \hfill \\ \end{gathered}$$

(C) {[*N*_*i*_(*n*_0_)]_0_ − [*N*_0_(*n*_0_)]_0_} is subtracted from [*N*_*i*_(*n*)]_*k*≥1_ in the rest of the segments, where *n*_0_ is the last coincidence event ID number in the first time segment. After this process, the following new numerical sequence is generated:

13 $$\begin{gathered} \left[ {N_{i} \left( n \right)} \right]_{{k \ge {1}}} \hfill \\ = \left\{ {\left[ {N_{i} \left( n \right)} \right]_{{1}} - \left\{ {\left[ {N_{i} \left( {n_{0} } \right)} \right]_{0} - \, \left[ {N_{0} \left( {n_{0} } \right)} \right]_{0} } \right\}, \, \left[ {N_{i} \left( n \right)} \right]_{{2}} - \left\{ {\left[ {N_{i} \left( {n_{0} } \right)} \right]_{0} - \, \left[ {N_{0} \left( {n_{0} } \right)} \right]_{0} } \right\}, \ldots } \right\} \hfill \\ \end{gathered}$$

(D) {[*N*_*i*_(*n*)]_1_ − [*N*_0_(*n*)]_1_} is subtracted from all elements of the numerical sequence within the second time-segment. After this process, the following numerical sequence is generated as a new time segment in this manner:

14 $$\begin{gathered} \left\{ {0, \, \left[ {N_{i} \left( {n_{0} + {1}} \right)} \right]_{{1}} - \, \left\{ {\left[ {N_{i} \left( {n_{0} + {1}} \right)} \right]_{{1}} - \, \left[ {N_{0} \left( {n_{0} + {1}} \right)} \right]_{{1}} } \right\}, \, \left[ {N_{i} \left( {n_{0} + {2}} \right)} \right]_{{1}} - \, \left\{ {\left[ {N_{i} \left( {n_{0} + {2}} \right)} \right]_{{1}} - \, \left[ {N_{0} \left( {n_{0} + {2}} \right)} \right]_{{1}} } \right\}, \ldots } \right\} \hfill \\ = \left\{ {0, \, \left[ {N_{0} \left( {n_{0} + {1}} \right)} \right]_{{1}} , \, \left[ {N_{0} \left( {n_{0} + {2}} \right)} \right]_{{1}} , \ldots } \right\} \hfill \\ \end{gathered}$$

(E) {[*N*_*i*_(*n*_1_)]_1_ − [*N*_0_(*n*_1_)]_1_} is subtracted from [*N*_*i*_(*n*)]_*k*≥2_ in the rest of the segments, where *n*_1_ is the last event ID number in the second time segment. After this process, the following new numerical sequence is generated:

15 $$\begin{gathered} \left[ {N_{i} \left( n \right)} \right]_{{k \ge {2}}} \hfill \\ = \left\{ {\left[ {N_{i} \left( n \right)} \right]_{{2}} - \left\{ {\left[ {N_{i} \left( {n_{{1}} } \right)} \right]_{{1}} - \, \left[ {N_{0} \left( {n_{{1}} } \right)} \right]_{{1}} } \right\}, \, \left[ {N_{i} \left( n \right)} \right]_{{3}} - \left\{ {\left[ {N_{i} \left( {n_{{1}} } \right)} \right]_{{1}} - \, \left[ {N_{0} \left( {n_{{1}} } \right)} \right]_{{1}} } \right\}, \ldots } \right\} \hfill \\ \end{gathered}$$

**Figure 5:**
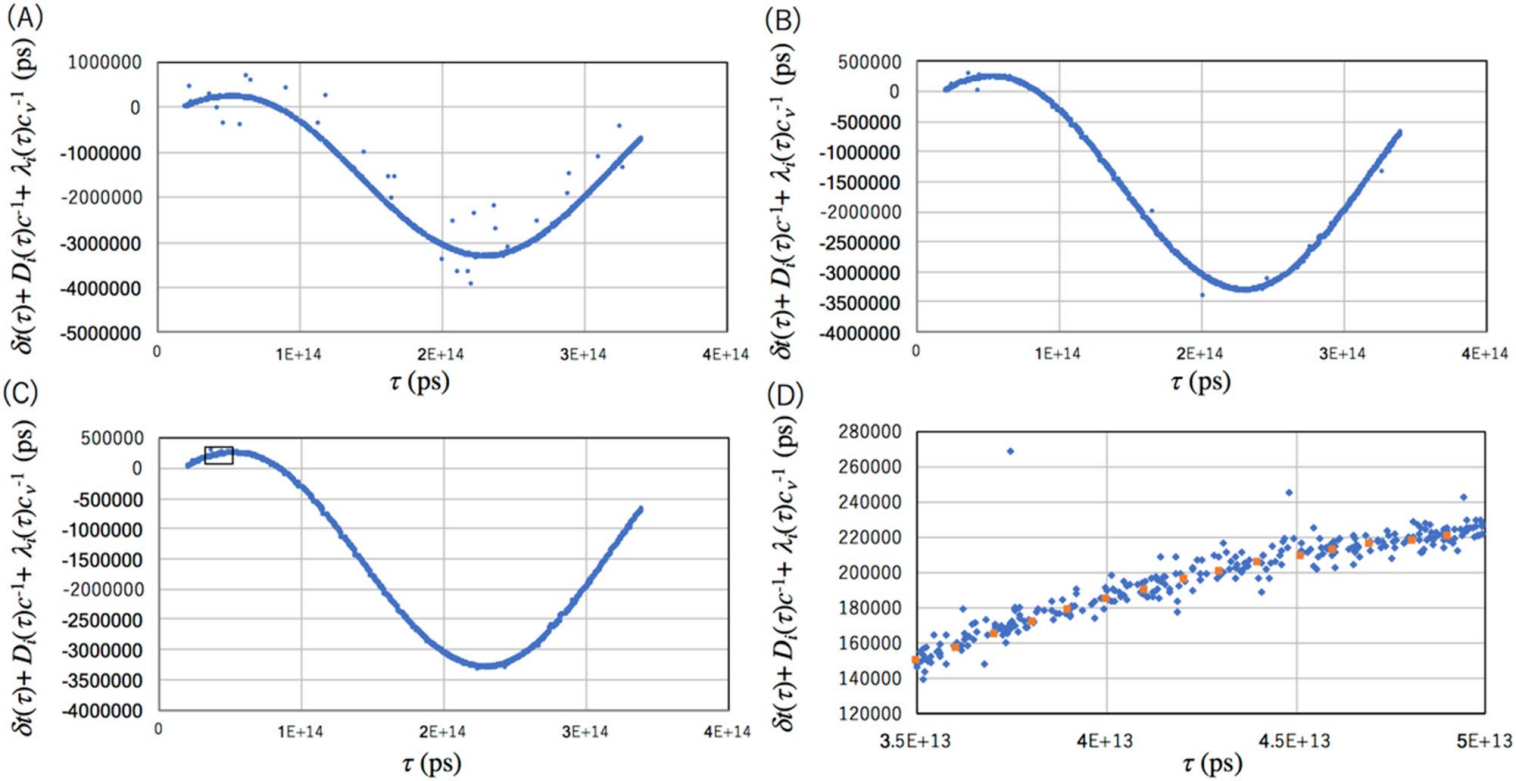
Retrodiction results. Results of the local clock drift with different coincidence time windows: (**A**) *T*_w_ = 1000 ns, (**B**) *T*_w_ = 300 ns, and (**C**) *T*_w_ = 100 ns are shown. The box in (**C**) indicates the temporal region of the magnified view shown in (**D**). Blue filled circles and orange filled circles respectively indicate the retrodiction results based on CTC and GPS-1PPS.

**Figure 6:**
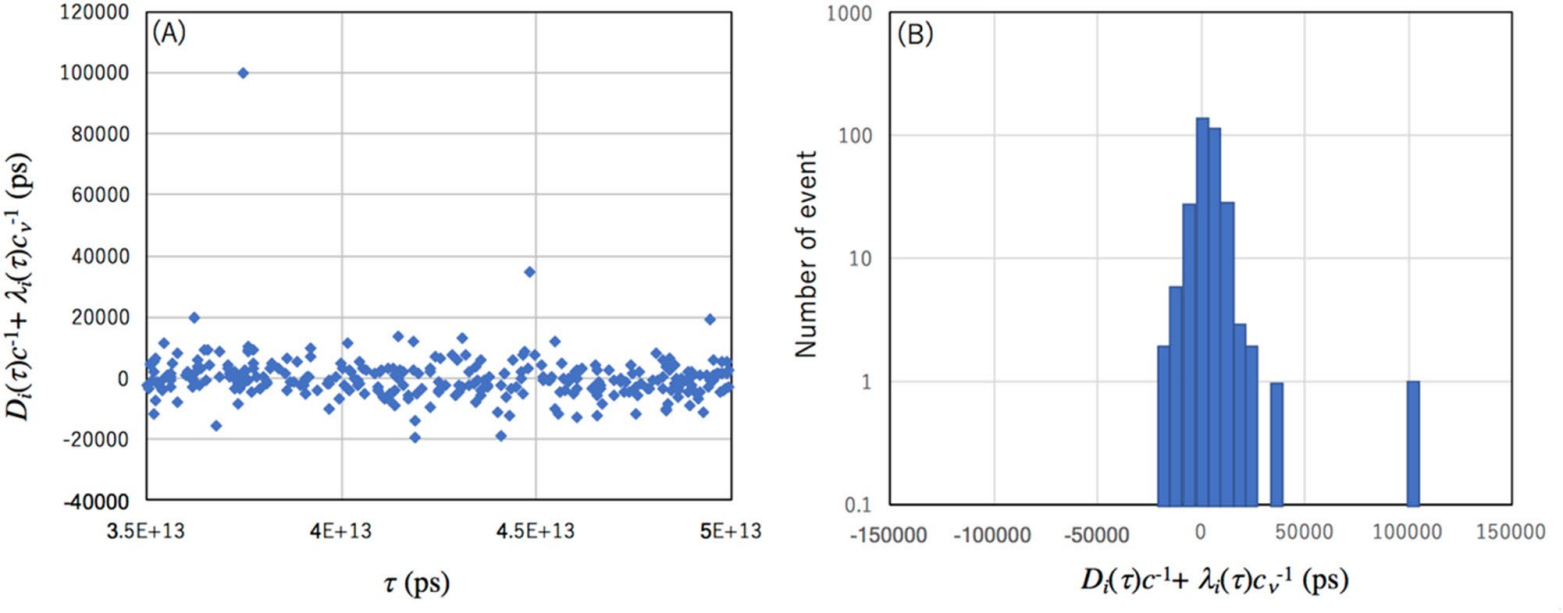
Plot where the artificial drift is subtracted from the data and its distribution. The data corresponds to Fig. 5D (**A**) and the distribution is shown in a logarithmic scale (**B**).

By repeating the processes from (A) to (F), the width of *T*_W_ does not need to expand; hence *f*_ACCIDENT_ can be suppressed. If the timelines are not divided into many segments, *T*_W_ needs to be widened so that it can cover the larger $\delta$ *t*. The online process from Eqs. (12) to (15) enables real-time resynchronization of the local clocks. Offline analysis from Eqs. (12) to (15) enables retrodiction. In Fig. 5, the results obtained with the current setup is shown. The coincidence rate was 60 Hz. Figure 5A–C respectively show the reconstructed value of *t*($\tau$) after being processed for *T*_W_ = 1,000 ns, 300 ns, and 100 ns. In these figures, increasing *T*_W_ increases *f*_ACCIDENT_ and thus, the dispersion around the expected value can be seen. 100 ns is a reasonable time window for *T*_w_. In the current artificial drift model, the clock drifts by 100 ns in 20 s at the maximum, therefore the length of the time segment was chosen to be 1 s, a value that is much shorter than 20 s. In a magnified view shown in Fig. 5D, fluctuations in [*D*_*i*_($\tau$)*c*^−1^ + $\lambda$_*i*_($\tau$)$c_{\nu }$^−1^] can be seen. The resynchronization error is ± 4.3 ns (S.D.). Figure 6A shows a plot where the artificial drift is subtracted from the data. Figure 6B introduces non-gaussian dispersion in the data. Accidental coincidence events can be seen as a deviation from the distribution. As a result of this prototype experiment, it has been shown that the current experimental setup is directly applicable to WSN for accurate retrodiction of events recorded in each sensor.

## Discussion

### Limits on the inter-nodal distance

For each given detector, *T*_W_ has to be narrower as the distance between Detector 0 and Detector *i* increases. As can be seen in Eq. (6), $f_{\upmu }$ decreases as this distance increases (in proportion to $\Omega$_*i*_) while *f*_ACCIDENT_ is independent from this distance. On the other hand, the minimum value of *T*_W_ depends on the timescale and the magnitude of $\delta$ *t*($\tau$). From Eq. (5), the tracking rate is approximated as follows:

16 $$\begin{gathered} f_{\upmu } = {\Phi }_{i} \sim {\Phi }S_{0} {\Omega }_{{\text{i}}} , \hfill \\ \sim {\Phi }S_{0}^{{2}} D_{i}^{{ - {2}}} , \hfill \\ \end{gathered}$$

for small $\Omega$ and *S*_0_ = *S*_*i*_. Equation (16) can be derived for *S*_0_ <  < *D*_*i*_^2^ since the solid angle is approximated to be *S*_0_cos $\theta$* D*_*i*_^−2^, where cos $\theta$ ~1 for $\theta$ < < 1. Therefore, the resynchronization error rate will be:

17 $$f_{{{\text{ACCIDENT}}}} f_{\upmu }^{{ - {1}}} = {2}T_{{\text{w}}} D_{i}^{{2}} .$$

$\delta$*t*($\tau$) is generally much larger than *D*_*i*_*c*^−1^, but in order to find the coincidence events between Detector 0 and Detector *i* which are used to track muons, *T*_w_ needs to be sufficiently larger than $\delta$*t*($f_{\upmu }$^−1^). Therefore, the limits of the inter-nodal distance are:

18 $$D_{i}^{{2}} < < { 1}/{2}f_{{{\text{ACCIDENT}}}} f_{\upmu }^{ - 1} {\Phi }^{ - 1} [\delta t(f_{\upmu }^{{ - {1}}} )]^{{ - {1}}} .$$

For example, in order to suppress the fraction of accidental coincidence events to less than 10%, for *T*_w_ = 1000 ns, 100 ns, and 10 ns, the maximum inter-nodal distance should be respectively less than 22 m, 71 m, and 223 m. The relationship between *D*_*i*_ and $\delta$*t*($f_{\upmu }$^−1^) will be further discussed in the modeling studies of the next section which involve a possible future application of CTC to the SHM of an aircraft. A more practical CTC setup and experimental examples of $\delta$*t*($\tau$) will also be introduced in the next section.

### Applications of CTC

**Figure 7:**
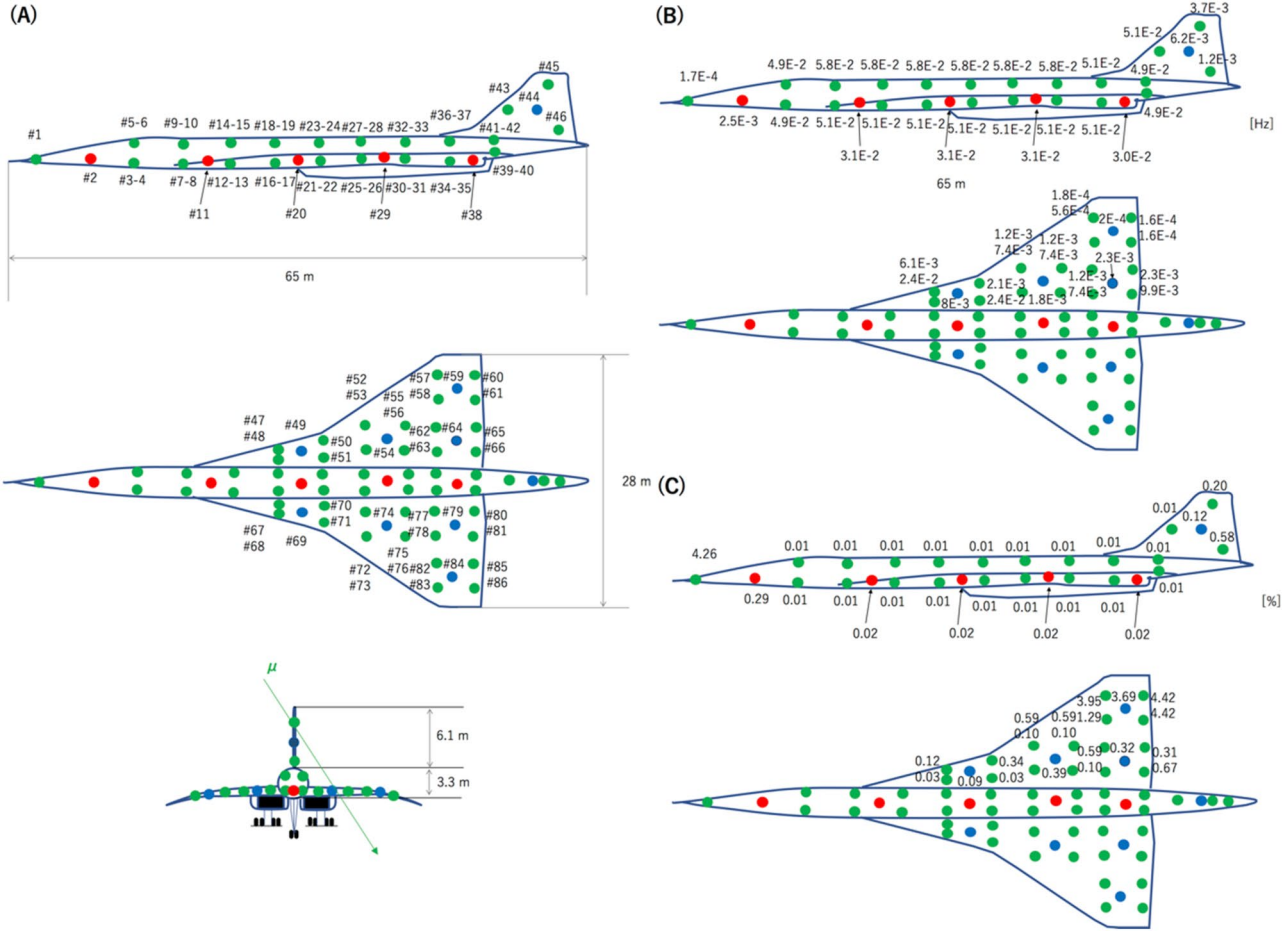
Intelligent health monitoring of aerospace structures using wireless sensor networks proposed by Yuan (NSF Award 0329878). While the sensor-node arrangement follows the Yuan's proposal, the Concorde was chosen as an aircraft body in this work. The red filled circles and blue filled circles respectively indicate master sensors located inside a cabin and wings. The green filled circles indicate the slave sensors. The identification numbers of the sensors are indicated in (**A**). The calibration frequency is shown in units of Hz in (**B**). The resynchronization error rate is shown in (**C**).

CTC is applicable to both real-time monitoring and offline analysis. The application of CTC to SHM of a Concorde-type airplane is modelled with a simulation to demonstrate and describe its capabilities. The geometrical configuration of the sensor nodes (Fig. 7A) used in the current modeling corresponds to the one proposed by the United States’ National Science Foundation (NSF Award 0329878) funded “Intelligent Health Monitoring of Aerospace Structures Using Wireless Sensor Networks” project which focused on development of a multi-sensor data fusion information framework for aerospace structural health monitoring (SHM) using smart sensors^25^. The identification numbers of the sensor nodes in this work are indicated by the symbol #. In these model studies, it was assumed that each detector associated with the sensor nodes can be used interchangeably, playing the role of either Detector 0 or Detector *i*, so that each time it is required, each sensor node can be resynchronized with the other sensor nodes. All the detectors would have this same flexible characteristic.

**Figure 8:**
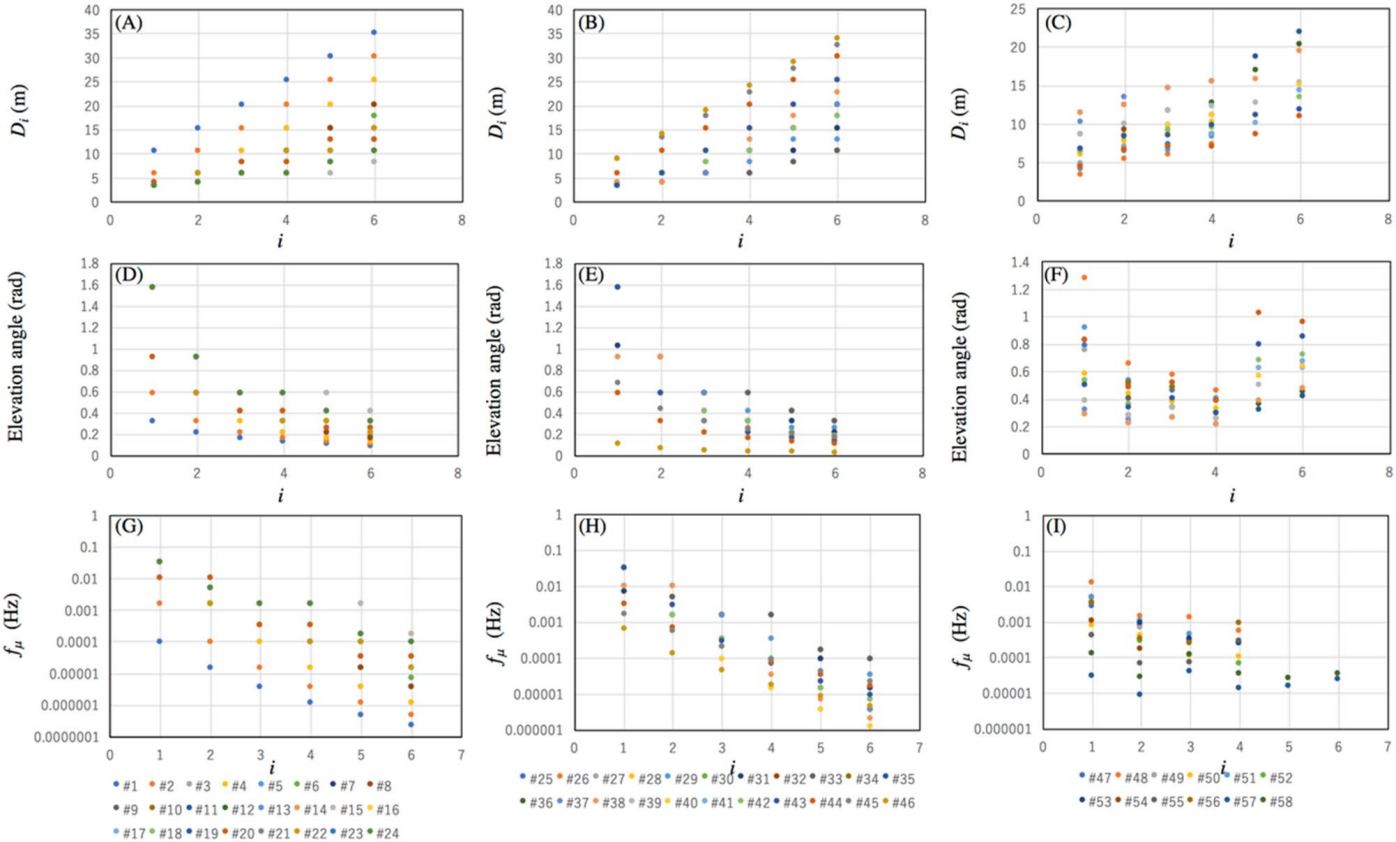
Distribution of inter-nodal distances (**A**–**C**), inter-nodal elevation angles (**D**–**F**), and resynchronization frequency at each node (**G**,**H**) based on the current geometrical configuration of the wireless sensors.

Figure 8A–C show *D*_*i*_ measured from each sensor node, where *i* numbers start from closer detectors to further detectors. Figure 8D–F shows the *i*-dependent elevation angles ($\theta$_*i*_) formed by Detector 0 and Detector *i*. As a general trend, as *i* increases, *D*_*i*_ increases and the elevation angle decreases. In the current modeling, *D*_*i*_ and $f_{\upmu }$ have the following relationship:

19 $$\begin{gathered} f_{\upmu } \sim \, \left[ {\mathop \sum \limits_{i}^{{}} {\Phi }(\theta_{i} )S_{0} {\Omega }_{i} } \right] \hfill \\ \sim [\mathop \sum \limits_{i}^{{}} {\Phi }(\theta_{i} )S_{0}^{{2}} D_{i}^{{ - {2}}} ], \hfill \\ \end{gathered}$$

where *S*_0_ corresponds to the size of an A4 piece of paper (600 cm^2^). As can be seen in Fig. 8G–I, $f_{\upmu }$ is exponentially reduced as a function of *i*. Therefore, for simplicity, summation in Eq. (19) was taken over by four neighbor detectors (*i* ≤ 4). To plot these figures, the position of the muon detectors was considered to take into account the muon flux anisotropy. The distances between sensors (Fig. 8A–C) angles formed by these sensors (Fig. 8D–F) are used for calculating the integrated muon flux (Fig. 8G–H), By substituting *S*_0_ to Eq. (7), we obtain *f*_ACCIDENT_ = 7.2 × 10^–6^ Hz for *T*_W_ = 100 ns. With these parameters, the calibration frequency and the resynchronization error rate were computed and the results are shown in Fig. 7B,C. $f_{\upmu }$^−1^ ranges from 17 s to 6,250 s, and the accidental-coincidence-based resynchronization error rate ranges from 0.01 to 4.26%.

**Figure 9:**
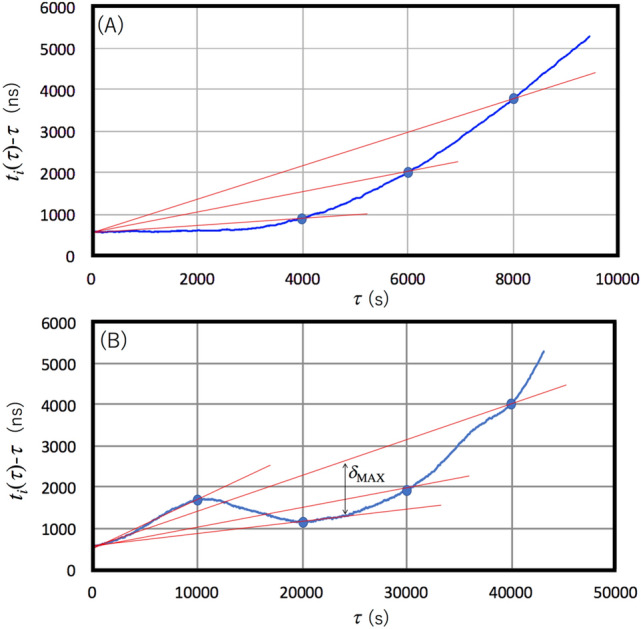
Drift curves measured with Trimble Thunderbolt GM200 OCXO (blue curves). The first (**A**) and second (**B**) strongest drift curves among 12 runs. Red lines indicate linear interpolations between blue filled circles. The maximum deviation between these red lines and the blue curves defines $\delta$_MAX_.

Now we evaluate the resynchronization accuracy attainable with the commercially-available OCXO in order to check that clocks do not drift too much given the low coincidence frequency or any other reason. We assumed the local clocks associated with the sensor nodes are equivalent to the OCXO (Trimble Thunderbolt GM200). Figure 9 shows two examples of $\delta$*t*−$\tau$ plots (as introduced with Fig. 1) measured with the OCXO. In this plot, Non-GPS-disciplined Trimble Thunderbolt GM200 (in the holdover mode) was measured with a reference to another GPS-disciplined clock (Trimble Thunderbolt GM200). The two strongest drift curves among 12 runs were exploited and plotted in Fig. 9. One of the drift rates reached a rate of 5 microseconds in less than 3 h, and the other reached a rate of 5 microseconds in 12 h. Since the official drift rate of this kind of OCXO is ± 1.5 s$\upmu$ (S.D.) per 4 hours^26^, these examples show what can be expected from (nearly) the worst-case scenarios. As shown in these figures, the general trend is that the retrodiction accuracy degrades more as the $f_{\upmu }$^−1^ increases. In Fig. 9A, $\delta$_MAX_ = 50 ns, $\delta$_MAX_ = 200 ns, $\delta$_MAX_ = 700 ns, and $\delta$_MAX_ = 1200 ns for $f_{\upmu }$^−1^ = 2,000 $f_{\upmu }$^−1^ = 4,000, $f_{\upmu }$^−1^ = 6,000, and $f_{\upmu }$^−1^ = 8,000 s. In Fig. 9B, $\delta$_MAX_ = 900 ns, $\delta$_MAX_ = 800 ns, and $\delta$_MAX_ = 1200 ns for $f_{\upmu }$^−1^ = 20,000, $f_{\upmu }$^−1^ = 30,000, $f_{\upmu }$^−1^ = 40,000 s. As a consequence, in our SHM modeling work assuming a Concorde-shaped aircraft body and Yuan's sensor configuration, it was found that the rate of $\delta$_MAX_ is less than 50 ns for the majority of the sensor nodes (with the exception of the sensor nodes located on the top of the cabin and on the tip of the wings ($\delta$_MAX_ = 700 ns)).

Another application of CTC includes the time calibration of ocean bottom seismometers (OBSs). OBSs also use local clocks for time synchronization, however, these clocks drift by a few seconds during a one-year observation^27^. Considering the P-wave velocities of ~ 6 km^−1^, a few seconds lead to a positioning error of 20 km. Since we know the exact location of the OBSs, if the detector is brought above the OBSs, the local clocks of these OBSs can be calibrated to GPS time. For example, an oceanographic research vessel called Chikyu measures 210 m in length and 38 m in width. Therefore, we can deploy a 100-m^2^ detector on a boat. We assume 1-m^2^ detector which is connected to the OBS located on the seafloor. It will be shown that the coincidence rate is much higher than the accidental rate as follow. Detector 0 with a size of 10 × 10 m^2^ is installed on the boat. In this discussion, Detector 0 was hypothetically installed to Chikyu. The size of Detector 1 associated with OBS is assumed to be 1 × 1 m^2^. The OBS is located at 100 m below sea level. Energies of the muons that can pass through 100-m water must be higher than 30 GeV. The solid angle formed by Detector 0 and Detector 1 which are vertically aligned with a vertical distance of 100 m is 10^–2^ sr. Therefore, the coincidence rate ($f_{\upmu }$) is expected to be ~ 10^–2^ Hz between these detectors. If the waterline area is simply approximated to (length) × (width), the Chikyu's waterline area is 8,000 m^2^. Considering the weight of this boat of 56,000 tons, the average thickness of this boat is 7 m water equivalent. Therefore, if the detector is located at the bottom of the boat, the muon flux is reduced to ~ 50 Hz. The single counting rate of Detector 0 and Detector *i* are respectively ~ 5 × 10^3^ Hz and ~ 1 Hz. Therefore, from Eq. (7), the accidental rate (*f*_ACCIDENT_) is calculated to be ~ 10^–3^ Hz for *T*_w_ = 100 ns, which is one order of magintude smaller than the coincident rate.

We conclude from these modeling experiments that by utilizing the unique characteristics of naturally abundant cosmic ray muons as probes for precise resynchronization of the local clocks associated with WSN, significant improvements in the accuracy of the SHM system as a whole and robustness of sensor nodes in particular will be possible. In addition, the penetrative characteristics of cosmic ray muons will enable developers of SHM networks to expand the offline CTC technology to new, more remote or more hostile environments (e.g., underground facilities, oil rigs, etc.) that are inaccessible with inter-nodal cables, GPS or other RF-based technology. Although online CTC requires timing data to be shared in real time between sensor nodes, the synchronization accuracy is independent from the data sharing frequency and thus, accurate synchronization is possible in less ideal conditions, such as when using low-rate Wi-Fi. With its unique capabilities and adaptability, CTC can contribute to the development of new critical and useful applications of SHM in a wider range of environments.

## Data Availability

The datasets used and/or analyzed during the current study available from the corresponding author on reasonable request.
